# Enhancing Training Precision: Unveiling the Barbell Velocity’s Role in Tailoring the Resistance Load for the China Badminton Team

**DOI:** 10.5114/jhk/183444

**Published:** 2024-05-17

**Authors:** Jianing Cui, Jixiang Liu, Chunlei Li

**Affiliations:** 1 Department of Physical Education, Shanxi Normal University, Taiyuan, China.; 2 Department of Physical Education, Changzhi University, Changzhi, China.; 3 School of Strength and Conditioning Training, Beijing Sport University, Beijing, China.

**Keywords:** velocity-based training, load control, precision training

## Abstract

Velocity-based resistance training is a fundamental component of sports science, offering a systematic approach to investigating the load variables of resistance exercises. This research focused on assessing the load across various resistance exercises by examining the barbell velocity during the concentric phase. The study involved 11 male athletes representing the China badminton team, who underwent 1RM testing for bench press, hip thrust, back squat, and single leg press exercises and the maximum repetition testing at load intensities of 60%, 70%, 80%, and 90% of 1RM. Simultaneously, measurements were taken of the barbell’s concentric phase velocity during each exercise. The findings revealed a robust negative correlation between barbell velocity and load intensity. Furthermore, exercises engaging greater muscle strength displayed smoother fitting curves. Analysis of velocity loss rates indicated that the hip thrust exhibited a higher completion percentage compared to the back squat and the bench press. Similarly, the non-dominant leg press showed a higher completion percentage than the dominant leg press. The study emphasizes the significance of delineating barbell velocity distributions in resistance training involving large muscle groups, as well as the accurate determination of load intensity. Precise load determination can be facilitated by employing fitting curves derived from distinct movement patterns and varying load intensities. The utilization of velocity data offers a quantifiable approach to achieving targeted training outcomes.

## Introduction

Resistance training plays a pivotal role in enhancing multiple aspects of physical performance, encompassing increased muscle strength, hypertrophy, augmented power output, enhanced movement speed, elevated local muscle endurance, fortified neuromuscular function, and improved sports prowess (Chamera et al., 2023; Kraemer and Ratamess, 2004; Krzysztofik et al., 2023; Mikolajec et al., 2017; Ruiz et al., 2008; Suchomel et al., 2016). The China badminton team strategically incorporates resistance training into their regimen, engaging in 4–5 sessions per week during the season’s preparation phase. This frequency is subsequently adjusted to 3–4 sessions per week leading up to competitions, with further reductions in frequency during the actual competition phase (Li, 2016). Considering the entire annual training cycle, resistance training is consistently upheld at 3–4 sessions weekly, often scheduled on dedicated training days to bolster foundational physical fitness. Grounded in the training framework of the Chinese badminton team, this study centers on the meticulous design of the load management during resistance training sessions.

Accurate management of exercise loads stands as a fundamental requisite to ensure optimal athlete adaptation. Striking the right balance is imperative; excessive loading amplifies the risk of overtraining, while insufficient loading undermines the anticipated training outcomes. Precise training methodologies underscore the meticulous quantification of the load. Among the components that constitute load structure, load intensity and load volume remain the most contentious aspects. In conventional resistance training, employing metrics such as %1RM (one repetition maximum), XRM (maximum weight that can be lifted X repetitions), repetitions, and sets to characterize load intensity and volume exhibits limitations, often accompanied by operational safety concerns (Bompa and Buzzichelli, 2017; González-Badillo et al., 2017; Haff et al., 2016; Jordan et al., 2015). Addressing these challenges, velocity-based training emerges as a viable solution. By gauging barbell velocity to gauge load magnitude (Jovanovic and Flanagan, 2014; Yan et al., 2018), this approach facilitates load quantification, promotes precise load application, and empowers athletes to achieve qualitative performance enhancements. This pursuit aligns with preparations for the upcoming 2024 Paris Olympics, where the Chinese badminton team draws from the successful lessons of the Tokyo Olympic Games (Chen, 2018). Nevertheless, research on the interplay between barbell velocity and the load within distinct resistance training methodologies remains scarce, especially in certain sports (De Hoyo et al., 2021; García-Ramos et al., 2018; Rebelo et al., 2023). Within the athletes’ training domain, this study delved into elucidating the correlation between barbell velocity and the load across diverse badminton-related resistance training modalities. Through experimental investigations involving professional badminton athletes, this research aimed to establish a robust framework guiding the accurate implementation of resistance training protocols.

## Methods

### Participants

The study cohort comprised 11 male athletes affiliated with the China badminton team, each holding a position within the top 100 rankings as recognized by the International Badminton Federation. All participants were screened to ensure their absence of sports-related injuries or health complications. Rigorous dietary control was implemented to maintain consistency across the participants’ nutritional intake. Prior to participation, athletes received comprehensive explanations regarding the study’s objectives, protocols, and possible associated risks. Subsequently, they provided informed consent through formal documentation. This study was conducted following the principles of the Declaration of Helsinki, and approved by the Beijing Sport University Ethics Committee of Sport (approval code: BSUECS2019091402; approval date: 14 September 2019). Additional information pertaining to participants is presented in Table 1.

**Table 1:** Basic information of participants.

N	Age (y)	Body height (cm)	Body mass (kg)	Resistance training experience (y)	Rank
12	21.3 ± 2.1	180.3 ± 4.3	75.0 ± 5.7	5.8 ± 0.9	<100

### Design and Procedures

All training sessions were conducted within the Physical Training Center of the General Administration of Sport of China and were scheduled at consistent times across different days. To ensure standardized conditions, participants refrained from engaging in strength training activities for two days preceding the tests. Prior to each test session, participants underwent a structured warm-up. This included a 10-min comprehensive relaxation routine employing a foam roller, followed by five joint flexibility exercises, three gluteal muscle activation exercises, and four dynamic stretching exercises. The uniformity of completed repetitions and the total practice duration of 25 min were maintained throughout the study period.

### Bench press (BP) in Badminton

In the realm of men’s badminton competitions, the smash stands as the technique yielding the highest scoring rate. Research has shown that the power of the upper limbs is positively correlated with the smash speed of badminton, while the smash involves internal rotation and adduction of the shoulder and extension of the elbow, and the bench press helps to accomplish the above actions (Sakurai et al, 2000). The bench press maneuver represents a crucial element integrated within the upper body strength training regimen for the national badminton team. As a norm, the training schedule typically entails 2–3 bench press sessions per week (Li, 2016).

### Hip thrust (HT) in Badminton

The hip thrust exercise is renowned for its ability to comprehensively enhance the hip extensors’ strength (Contreras et al., 2011). It notably contributes to elevating horizontal force generation while concurrently mitigating the susceptibility to hamstring injuries (Hoskins and Pollard, 2005). By augmenting the mechanical efficacy of lower limb actions, the hip thrust further curtails the occurrence of waist and hamstring-related injuries. This exercise holds a position of prominence among badminton players, who frequently incorporate it into their training routines. Depending on the training phase of the badminton team, hip thrust sessions are strategically scheduled 1–2 times per week.

### Back squat (BS) in Badminton

The back squat stands as a quintessential training technique for cultivating lower body strength, yielding substantial enhancements in the execution of pivotal movements such as initiating, changing direction, and stepping. Within the realm of the China badminton team, both coaches and athletes underscore the significance of integrating back squat exercises. As a common practice, the badminton team dedicates 2–3 weekly sessions to back squat workouts, recognizing the pivotal role of this exercise in their training regimen.

### Single leg press in Badminton

The seated leg press holds a prominent position in the training repertoire of the China badminton team. This exercise finds particular favor among team members grappling with waist injuries, presenting an optimized training approach tailored to their needs. Given the prevalence of one-sided racket-holding dynamics in badminton, the single leg press holds distinct significance, warranting 1–2 weekly sessions. Athletes inherently possess a dominant and a non-dominant side, each corresponding to their racket-holding and non-racket-holding roles, respectively. Research underscores that the dominant leg aligns with the racket-holding function, while the non-dominant leg pertains to the opposite role. Subsequently, the single leg press was further delineated into the non-dominant leg press (NDLP) and the dominant leg press (DLP), emerging as two distinct exercises for in-depth exploration.

In alignment with the prescribed guidelines for 1RM testing and tailored to the coaches’ and athletes’ specific circumstances, the five resistance training exercises (including the non-dominant leg press and the dominant leg press) were executed with distinct initial weights, incremental protocols, and interval time, all of which were modulated based on the barbell velocity. As the velocity reached a designated threshold, participants were directed to execute a singular repetition (Gary et al., 2018; Kraemer et al., 2004). Further details are outlined in Table 2. All of the above test movements used a time ratio of 2:1 between the eccentric and concentric phases, with no pause in the transition between the two phases, and there was continuous encouragement throughout the phase, requiring the lifting to be as fast as possible (Lu et al., 2023; Tsoukos and Bogdanis, 2023). The 1RM test was completed in three classes, with the 1RM test for the bench press and the hip thrust in the first class, the 1RM test for the non-dominant leg press and the dominant leg press in the second class, and the 1RM squat test in the third class, with a minimum 30-min rest interval between the 1RM tests for the two exercises in the same class. The exercises and procedures were standardized according to the guidelines issued by the National Strength and Conditioning Association.

**Table 2:** 1RM test parameters for different movements.

Movement	Initial weight (kg)	Incremental weight (kg)	Interval time (min)	Velocity remind
BP	60	10	3–5	≥0.5 m/s, 2 reps <0.5 m/s, 1 rep
HT	120	20	4–6	≥0.6 m/s, 2 reps <0.6 m/s, 1 rep
BS	100	10	4–6	≥0.5 m/s, 2 reps <0.5 m/s, 1 rep
NDLP	50	10	3–5	≥0.55 m/s, 2 reps <0.55 m/s, 1 rep
DLP	60	10	3–5	

Individual peak strength levels were ascertained via the 1RM test, followed by a comprehensive exploration of the distinct characteristics inherent to five movement patterns. According to the long-term arrangement of resistance training of the China badminton team, the load intensity was generally greater than 60%1RM. Subsequently, the maximum repetition experiment was conducted across various load intensities, namely 60%1RM, 70%1RM, 80%1RM, and 90%1RM (Rodríguez-Rosell et al., 2019). Each participant completed a total of 20 testing sets (5 exercises x 4 load intensities). To mitigate any potential influence of sequence bias, testing order was randomized, incorporating two sets of maximal repetitions within each session. A minimum interval of 10 min separated the two sets to prevent any detrimental impact on experimental outcomes (Gentil et al., 2018). These testing sessions were scheduled 2–3 times per week.

The most critical velocity monitoring devices include the GymAware which synchronizes the barbell velocity for each movement. The GymAware is attached to the weight plate on the floor in order to ensure that the string is perpendicular to the barbell trajectory. In our study, mean velocity data collection involved employing GymAware PowerTool (#3645, Australia) which is highly accurate and is the only linear position transducer offering x-axis calibration to capture valid velocity data during motion.

### Statistical Analysis

This paper adopts a methodology centered on exploring velocity-based resistance training with a focus on badminton athletes. The velocity indicator analysis was carried out using Origin 2021 and SPSS 26.0 software for statistical and graphical analyses. This was done by making scatter plots of load intensity versus barbell mean velocity, the percentage of repetitions completed versus the velocity loss rate, and analyzing them with linear regression. Both continuous variables were normally distributed, thus a robust inverse correlation between barbell mean velocity and load intensity emerged across the diverse movements through a Pearson’ correlation test, underscoring the imperative of meticulous velocity control (p < 0.01). Furthermore, curvilinear regressions were performed on the percentage of repetitions completed and the velocity loss rate under different load intensities. Then multiple function models were selected, and after adaptation, the quadratic function had the best fit (p < 0.01). These insights lay the foundation for precision load quantification, with far-reaching implications for optimizing training methodologies in badminton and beyond.

## Results

### Relationships between Load Intensity and Barbell Velocity

The correlation’s validation between load intensity and barbell mean velocity within the context of badminton remains uncharted. The China badminton team has embarked on an investigation into this interrelation, striving to elucidate the connection between load intensity and barbell velocity. The utilization of barbell velocity data holds the potential to serve as a guiding metric, enabling coaches to precisely calibrate resistance training intensity. This practice stands poised to facilitate the formulation of more accurate and effective resistance training protocols.

**Figure 1:**
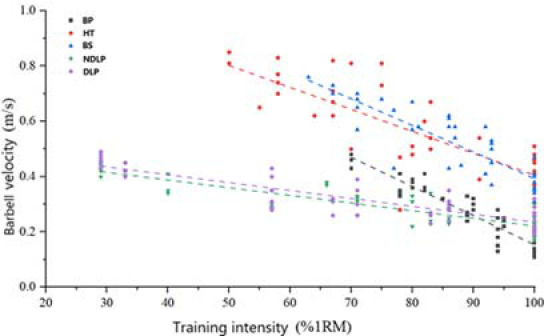
The relationship between barbell velocity and load intensity against different exercises.

The amassed GymAware data underwent rigorous analysis and fitting using Origin 2021, revealing distinct relationships between load intensity and barbell velocity across the five movements. These findings are depicted in Figure 1. The correlations were as follows: for the bench press, the equation was y = 1.175 – 1.0083x, with an R^2^ value of 0.943; for the hip thrust, it was y = 1.201 – 0.7726x, with an R^2^ value of 0.949; the back squat was represented by y = 1.3624 – 0.9721x, boasting an R^2^ value of 0.968; for the non-dominant leg press the equation was y = 0.5229 – 0.3206x, with an R^2^ value of 0.945; and for the dominant leg press, it was y = 0.5368 – 0.2996x, with an R^2^ value of 0.933. Notably, all the fitting functions yielded R^2^ values exceeding 0.9, indicative of robust linear relationships.

Among the bilateral exercises examined within this experiment, the bench press exhibited the steepest slope in its fitting function. This implies that the barbell velocity during the bench press experienced the most pronounced reduction as the unit load intensity increased. In contrast, the fitting function slope for the hip thrust was the most gradual, indicating that within a narrower range of motion, which typically demands greater muscular strength, the decline in barbell velocity was less substantial with heightened load intensity. Notably, at an 80.9% 1RM load intensity, the barbell velocity during the hip thrust equaled that of the back squat. However, as the load intensity surpassed 80.9%1RM, the barbell velocity during the hip thrust was greater compared to that of the back squat, emerging as the highest among all five exercises.

Concerning unilateral leg exercises, the slope of the fitting function for the non-dominant leg press was marginally steeper than that of the dominant leg press. Despite this variation, statistical analysis demonstrated no significant difference between the two. Moreover, the constant of the dominant leg’s function slightly surpassed that of the non-dominant leg. Given the dominant leg’s superior strength, as load intensity elevated, the reduction in lifting velocity became less pronounced in the dominant leg. Consequently, under identical load intensity, the barbell velocity of the dominant leg outpaced that of the non-dominant leg. Within the context of unilateral exercises, the side exhibiting greater strength manifested a more gradual decline in barbell velocity as load intensity increased.

Employing various load intensities in training contributes to distinct categories of strength. Additionally, insights derived from the correlation between load intensity and barbell velocity highlight that enhancing diverse strength attributes necessitates varying barbell velocity profiles. Through the combination of the aforementioned fitting functions, a comprehensive understanding of the interrelation between the development of diverse strength qualities and barbell velocity may be established.

Research findings indicate that engaging in training with load intensities ranging from 5% to 25% of 1RM fosters the development of an individual’s activation force. Conversely, employing load intensities of 85%1RM and above contributes to the enhancement of absolute strength. In accordance with the strength-speed continuum, the domain of 1–1.5 m/s effectively contributes to the enhancement of speed-strength capabilities. Within this experiment, the optimal barbell velocity zones for improving body speed-strength were identified for the bench press (0.72–0.92 m/s) and the back squat (0.92–1.12 m/s). Discrepancies in the reported data stem from variations in experimental testing methodologies. This experiment employed distinct load intensities across various movement modes, yielding regression equations grounded in experimental load intensity and velocity. Consequently, these regression equations possess a defined scope of applicability.

### Correlation between Load Volume and the Velocity Loss Rate

During the maximum repetitions experiment, it was evident that as the completion count rose, fatigue experienced by participants increased, leading to a corresponding decline in the velocity of each movement. Quantified by the velocity loss rate (= 1 – movement velocity/fastest velocity in the set), the reduction in barbell velocity could serve as an indicator of both the fatigue level of participants and their overall physical condition. This offers an improved means to monitor participants’ well-being. It is imperative to recognize that once the velocity loss rate reaches a specific threshold, persisting with training can lead to diminishing training effects (Baker and Newton, 2007; Pareja-Blanco et al., 2017). As a result, comprehending the reality of the velocity loss rate across different load intensities becomes paramount. Equally critical is identifying the point at which this rate reaches a significant value, indicating the conclusion of the training volume. The integration of the percentage of repetitions completed, as a replacement for the load indicated by completed repetitions, affords a valuable perspective. Specifically, it reflects the proportion of repetitions completed in relation to the maximum attainable repetitions. Through an exploration of the correlation between the percentage of repetitions completed and the velocity loss rate across diverse load intensities, the number of repetitions to be accomplished can be judiciously determined. This approach permits an objective evaluation of each athlete’s specific condition, offering coaches a convenient tool to monitor training in accordance with the established velocity loss rate benchmarks.

**Figure 2:**
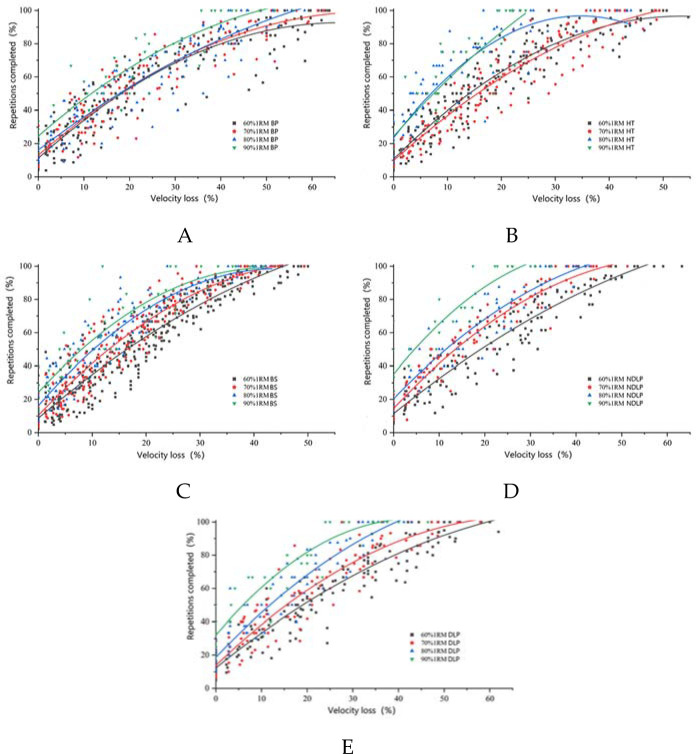
The relationship between the percentage of repetitions completed and the velocity loss rate against different load intensities (A: bench press; B: hip thrust; C: back squat; D: non-dominant side leg press; E: dominant side leg press).

Based on Figure 2, it becomes evident that the correlation between the velocity loss rate and the percentage of completed repetitions diverges significantly across varying intensities and movements. When training at 90%1RM, it requires the highest percentage of repetitions completed to achieve a comparable velocity loss rate, followed by 80%1RM, with the lowest percentage of completed repetitions occurring at the 60%1RM load. However, the same phenomenon is less pronounced in the hip thrust. This difference might be attributed to the substantial load encountered during the hip thrust, which could hinder precise control of barbell movement during the eccentric phase. This, in turn, may slightly alter the range of motion during the completion process, particularly when nearing maximum intensity. It is worth noting that in the sub-maximal intensity experiment, some participants experienced discomfort due to heightened pressure on the ilium and the abdomen, ultimately leading to compromised sports performance. Simultaneously, this exercise necessitates exceptional core stability and strength.

Table 4 provides a compelling perspective, revealing that R^2^ values of the fitting curves for all five exercise modalities at four load intensities surpassed 0.85 (excluding the 90%1RM hip thrust). This signifies a robust quadratic function relationship between the velocity loss rate and the percentage of completed repetitions. The point at which the percentage of repetitions reaches 100% indicates that the exercise set has reached the maximum exhaustion. Investigating the maximum velocity loss rate at this stage bears substantial practical importance, facilitating coaches and athletes in establishing a prudent safety threshold. As the load intensity increases from 60% to 100%1RM, there is a progressive reduction in the maximum velocity loss rate. Specifically, the maximum velocity loss rate ranged from 57.2 ± 10.9 to 43.3 ± 6.7 for the bench press, 45.5 ± 7.3 to 21.7 ± 1.6 for the hip thrust, 46.2 ± 3.5 to 31.9 ± 9.2 for the back squat, 50.5 ± 8.6 to 26.1 ± 4.9 for the non-dominant leg press, and 52.6 ± 7.4 to 32.9 ± 7.8 for the dominant leg press. Notably, among these exercises, the back squat yielded the highest maximal velocity loss rate, with the bench press following closely behind.

**Table 3:** The velocity zone of developing different types of strength under different movements.

%1RM	Strength Quality	Velocity Zone	Movements				
			BP	HT	SQ	NDLP	DLP
5–25	Activation force	>1.5	>*0.92*	>*1.01*	>*1.12*	>*0.44*	>*0.46*
25–45	Speed-Strength	1.0–1.5	*0.72*–*0.92*	*0.85*–*1.01*	*0.92*–*1.12*	0.39–0.44	0.40–0.46
45–65	Strength-Speed	0.75–1.0	0.52–0.72	*0.70*–*0.85*	0.73–0.92	0.31–0.39	0.34–0.40
65–85	Power	0.45–0.75	0.32–0.52	0.54–0.70	0.54–0.73	0.25–0.31	0.28–0.34
85–100	Absolute strength	<0.45	<0.32	<0.54	<0.54	<0.25	<0.28

Note: The content in italics and bold within the table represents estimated values derived from the regression equation. The velocity segments delineating strength training objectives are determined in alignment with the strength-speed curve, with all speed units standardized to m/s.

**Table 4:** Fitting equations of the percentage of repetitions completed and the velocity loss rate under different load intensities.

Movement	%1RM	Fitting equations	R^2^	F
BP	60	y = −0.0186*x^2^ + 2.4626*x + 11.2145	0.883	803.095
	70	y = −0.0162*x^2^ + 2.3645*x + 13.0667	0.883	551.005
	80	y = −0.0107*x^2^ + 2.4626*x + 16.1763	0.862	341.825
	90	y = −0.0169*x^2^ + 2.3741*x + 24.6163	0.869	156.335
HT	60	y = −0.0301*x^2^ + 3.2185*x + 10.6618	0.882	788.129
	70	y = −0.0224*x^2^ + 2.938*x + 9.652	0.890	645.441
	80	y = −0.0608*x^2^ + 4.2197*x + 23.5009	0.862	341.825
	90	y = −0.0246*x^2^ + 3.6214*x + 24.397	0.805	100.846
BS	60	y = −0.0172*x^2^ + 2.7925*x + 8.6593	0.887	1329.272
	70	y = −0.032*x^2^ + 3.4535*x + 10.2524	0.892	885.690
	80	y = −0.0422*x^2^ + 3.7296*x + 16.1202	0.888	477.187
	90	y = −0.0419*x^2^ + 3.5434*x + 24.1423	0.855	162.169
NDLP	60	y = −0.0111*x^2^ + 2.2286*x + 11.3584	0.892	584.864
	70	y = −0.0236*x^2^ + 2.9373*x + 14.4608	0.934	811.090
	80	y = −0.0608*x^2^ + 4.2197*x + 23.5009	0.885	276.924
	90	y = −0.039*x^2^ + 3.404*x + 35.0804	0.872	105.093
DLP	60	y = −0.0125*x^2^ + 2.2193*x + 12.2808	0.907	765.677
	70	y = −0.0195*x^2^ + 2.6477*x + 13.7169	0.879	407.636
	80	y = −0.0211*x^2^ + 2.8951*x + 18.6231	0.876	287.486
	90	y = −0.0375*x^2^ + 3.2422*x + 31.9191	0.880	135.979

Based on an analysis of training requisites, diverse load intensities are devised. Within these load ranges, the velocity loss rates are ascertained. Correspondingly, referencing Table 4 aids in establishing the percentage of repetitions completed, affording a systematic and precise understanding of the load embedded within each set. This approach ensures a scientific and accurate grasp of training loads, enabling exact control over training regimens.

## Discussion

### Establishment of the Load Intensity of Resistance Training

A robust linear association has been well-established between load intensity and barbell mean velocity in existing literature (González-Badillo et al., 2011; González-Badillo and Sánchez-Medina., 2010; Sánchez-Medina et al., 2014, 2017). However, most of the international research on velocity-based resistance training theories stems from a limited number of research groups. This concentration can potentially introduce authorial bias, underscoring the need for a broader array of scholars to engage in multidimensional research initiatives. Notably, most investigations into velocity-based training have primarily centered around Smith machine exercises, often confined to controlled laboratory settings (García-Ramos et al., 2019; González-Badillo et al., 2010; Pérez-Castilla et al., 2020). Unfortunately, the utilization of unilateral exercises remains relatively few (Liao et al., 2023; Martínez-Rubio et al., 2023), and the movement specifications adopted in these experiments often fall short of addressing real-world demands, not meeting the needs of unilateral program athletes. This study, however, differentiates itself by employing China badminton athletes as participants, zeroing in on the freestyle bench press, the hip thrust, and the back squat—integral components of the badminton team’s regular training regimen. The inclusion of unilateral movements involving the dominant leg serves to refine and optimize athletes’ unilateral lower body strength training strategies. Moreover, it serves to diversify the theoretical underpinnings and practical applications of velocity-based training methodologies.

García-Ramos et al. (2021) explored the correlation between average barbell velocity and load intensity during the bench press, revealing MV = −0.0156 × %1RM + 1.712 using the Smith machine. However, this paper delves into the freestyle bench press, encompassing a broader load range of 60–100%1RM, which yields subtly different findings. The conclusion drawn here is MV = −1.0083 × %1RM + 1.175. DeHoyo et al. (2021) conducted a study involving 102 amateur powerlifters, yielding MV = −0.0102 × %1RM + 1.2976 for the hip thrust. In contrast, this research assessed the hip thrust performance of 11 world-class athletes, revealing MV = −0.7726 × %1RM + 1.201. This discrepancy may stem from variations in the resistance training levels of participants. Pérez-Castilla’s research (2020) on the back squat established a relationship between load intensity and barbell velocity, expressed as MPV = −0.012 × %1RM + 1.465. Here, MPV represented mean propulsion velocity, significantly differing from the mean velocity (MV) used in this study. Additionally, their use of the Smith machine yielded a less steep reduction in barbell velocity with increasing unit load intensity. This research, conducted with the freestyle back squat, determined MV = −0.9721 × %1RM + 1.3624. Conceição et al. (2016) examined the barbell velocity across three movements, including the seated leg press, resulting in MPV = −0.0180 × %1RM + 1.969. This study, however, focused on the unilateral leg press with participants engaged in unilateral sports, thereby introducing an inherent dichotomy between dominant and non-dominant sides, leading to training disparities between the sides. The study’s findings demonstrate that for the non-dominant leg press, MV = −0.3206 × %1RM + 0.5229, while for the dominant side leg press, MV = −0.2996 × %1RM + 0.5368. When executing unilateral movements and monitoring velocity, it is crucial to meticulously account for the conditions of both sides.

Upon the initiation of the first movement within each set, coupled with the insights from Table 3, a precise assessment of the degree to which the load stimulates the athlete’s system becomes feasible. By adeptly managing the real-time barbell velocity within predefined velocity thresholds and leveraging velocity metrics to gauge load intensity, the cultivation of distinct strength attributes attains greater precision.

### Determination of the Load Volume of Resistance Training

Utilizing the number of training repetitions as a gauge for load assessment merely affords a rudimentary evaluation of training completion, falling short in elucidating the training’s intrinsic quality. Effective training necessitates the engagement of all motor units within each movement, thereby optimizing the impact on the neuromuscular system.

After establishing the load intensity in training, the meticulous control of load volume assumes heightened significance. Excessive load volume might impede athletes from achieving the prescribed load intensity, while insufficient load volume could fail to effectively stimulate skeletal muscle growth. In this study, the nexus between the percentage of completed repetitions and the velocity loss rate within the spectrum of 60–90% 1RM was rigorously investigated for the bench press, the hip thrust, the back squat, the non-dominant leg press, and the dominant leg press exercises. The outcomes unveiled a robust quadratic function correlation, enabling precise determination of load volume. As athletes executed each movement at maximal velocity, the augmentation in repetition count was linked to a diminishing velocity decline, accentuating the relevance of this relationship.

The findings from González-Badillo et al. (2017) revealed a robust positive correlation between the velocity loss rate and the percentage of completed repetitions for a given movement and load intensity (R^2^ = 0.83), akin to the outcomes observed in this study. This paper, specifically, establishes this relationship across five movements within the range of 60%–90%1RM. When considering the same movement and the same velocity loss rate, an increase in load intensity corresponds to an augmented percentage of completed repetitions. This increase is mirrored by the upward trajectory of the functional representation. Deviations from prior research findings may be attributed to the athletic prowess of participants. While many research groups employ non-athletic healthy males, this study encompasses elite badminton players. Insights garnered from Table 4 suggest that, under identical velocity loss rates, movements involving limited muscle engagement exhibit lower percentages of completed repetitions compared to movements engaging more muscle groups (Rodríguez-Rosell et al., 2020). Notably, this study underscores that under equivalent load intensity and velocity loss rates, the sequence of the percentage of completed repetitions follows the hip thrust > the back squat > the bench press, closely mirroring prior research. However, in the same context, for the non-dominant leg press, the percentage of repetitions completed was greater than that of the dominant side. This divergence may trace its roots to the technical attributes of badminton, where the dominant leg primarily handles jumping and crossing movements, while the non-dominant leg is more involved in pedaling actions—similar in structure to single leg press movements.

## Conclusions

Velocity-load relationship: A robust negative correlation was established between barbell velocity and load intensity across the spectrum of freestyle bench press, hip thrust and back squat exercises. This relationship held true for both dominant and non-dominant leg press exercises. Notably, movements requiring greater muscle strength exhibited smoother fitting functions. Precision in resistance training hinges upon meticulous attention to the optimal barbell velocity range.

Repetition completion and velocity loss: When performing bench press, hip thrust, back squat, non-dominant leg press, and dominant leg press exercises within the 60%–90%1RM range, a substantial quadratic function relationship was observed between the percentage of repetitions completed and the velocity loss rate. The disparities within this relationship were substantial. As the velocity loss rate increased, the increase in the percentage of repetitions completed was notably restrained. Moreover, uniform velocity loss rates exhibited greater percentages of repetitions completed as load intensity rose. Within this framework, the bench press manifested the lowest fitting image, with the hip thrust at the upper end and the back squat intermediate. Additionally, the non-dominant leg press surpassesed the dominant leg press. The determination of load volume should scrupulously align with the fitting curve data derived from distinct movements and varied load intensities.

Accurate load quantification: Leveraging the correlation between barbell velocity and load intensity, precise quantification of load became feasible. This allowed a greater level of accuracy of resistance training, and in consequence, its enhanced efficacy.

In summation, these research findings underscore the vital importance of comprehending the intricate interplay between velocity, load intensity, and repetition completion in of the design of precision-based resistance training methodologies.

## Practical Implications

Wider application of velocity-based training: This study offers a systematic approach for assessing load intensity and load volume within the badminton’s resistance training domain via velocity indicators. Empirical evidence from China badminton team players underscores the substantial enhancements in sports performance achievable through such exercises. As a result, the integration of velocity-based training should be a central consideration in the routine resistance training and competitive preparation protocols for professional badminton teams. Although the sample size selected for this experiment is very small, according to the force-velocity curve and the results of the study, it is known that there is a quantitative relationship between velocity and the load in resistance training. However, this relationship is different in different movement patterns. This study focused on five movement patterns, and except for the single leg press, findings from the other four exercises are applicable to the general population. In the present study, the load intensity range was within 60–100% 1RM, which basically meets the general requirements for developing strength, thus by determining training goals and choosing appropriate exercises, we can refer to the results of the present study to carry out resistance training. Given the overarching principles of this training methodology, its broad applicability across diverse sports warrants its widespread use.

Exploring optimal velocity loss rates: This study established the percentage relationship of completed repetitions based on the velocity loss rate as a standardized benchmark. This permits the determination of the necessary number of repetitions per set in alignment with the established velocity loss rate. However, the exact velocity loss rate that maximizes strength quality remains an open question. Future research should converge on investigating the impact of varying velocity loss rates on the efficacy of strength training. Collaborations among coaches, athletes, and scholars are pivotal in shedding light on this aspect and refining training methodologies.
